# Cardiac Arrest and Hypoxic-Ischemic Encephalopathy Following an Emergency Caesarean Section in a COVID-19 Patient

**DOI:** 10.7759/cureus.21867

**Published:** 2022-02-03

**Authors:** Sai Palati, Anthony Sanchez, Maryellen Campbell, Martin Castaneda

**Affiliations:** 1 Obstetrics and Gynecology, Florida Atlantic University Charles E. Schmidt College of Medicine, Boca Raton, USA; 2 Obstetrics and Gynecology, Bethesda Hospital East, Boynton Beach, USA

**Keywords:** covid-19, sar-cov-2 infection, severe acute respiratory syndrome coronavirus 2 (sars-cov-2), sars-cov-2 during pregnancy, coronavirus in pregnancy, covid-19 in pregnancy

## Abstract

While young, healthy individuals without underlying medical conditions have generally not suffered catastrophic health consequences from the severe acute respiratory syndrome coronavirus 2 (SARS-CoV-2), gravid patients appear to be at much higher risk of complications from this virus. A 29-year-old G3P2 patient at 30 weeks and three days presented with worsening dyspnea and chest pain after testing positive for coronavirus disease 2019 (COVID-19) infection two days prior. Notably, she had not received COVID-19 vaccination. A non-reassuring fetal tracing and fetal bradycardia were discovered on routine prenatal monitoring during admission, and an urgent caesarean section was performed. She subsequently required supplemental oxygen due to respiratory distress and remained hospitalized. She clinically deteriorated from a respiratory standpoint. Several days later, she experienced cardiac arrest with a return of spontaneous circulation (ROSC) in nine minutes. While the baby was discharged home and is doing well, the patient, unfortunately, expired from hypoxic encephalopathy secondary to COVID-19 pneumonia and complications of cardiorespiratory arrest. This case highlights the severe sequelae of COVID-19 infection in a postpartum patient, including ventilator-dependent respiratory failure, sudden cardiac death, hypoxic encephalopathy, and coma.

## Introduction

Severe acute respiratory syndrome coronavirus 2 (SARS-CoV-2) can cause severe complications in gravid patients due to respiratory disease from the virus in addition to physiological adaptations of pregnancy such as decreased functional reserve capacity [1]. Changes to the maternal immune system is one of the major physiological adaptations of pregnancy. The maternal immune system undergoes adaptations that allow for the growth of a semi-allogenic fetus within the mother. These changes can cause a difference in the maternal immune response to infections during pregnancy, particularly to SARS-CoV-2 [2]. During gestation, maternal levels of natural killer (NK) cells decrease [3]. NK cells are innate immune cells that are known to have cytotoxic activity against virally infected cells. Therefore, the pregnant woman’s ability to protect herself from viral infections is attenuated. Additionally, NK cells are reduced during infection with SARS-CoV-2 infection [3]. As a result, a COVID-19-positive gravid patient is at risk for acquiring additional respiratory viral infections. Additionally, during pregnancy, the maternal immune system prioritizes humoral immune responses over cellular responses, with the CD4+ T cell population being Th2 phenotypes rather than Th1 phenotypes [3]. This is especially important considering patients with severe outcomes of COVID-19 also displayed reduced numbers of adaptive immune cells relative to those who did not experience severe outcomes [4].

The combination of immunological adaptations during pregnancy along and the systemic effects of SARS-CoV-2 can theoretically worsen respiratory disease severity during pregnancy. The currently available data on COVID-19 infection in pregnancy is limited. Son et al. examined women who delivered before the global pandemic compared with those delivering after the global pandemic [5]. They did not find a significant difference in the frequency of preterm birth, stillbirth, small for gestational age, large for gestational age, hypertensive disorders of pregnancy, placental abruption, caesarean birth, or postpartum hemorrhage between gravid patients who tested positive for SARS-CoV-2 infection and those who tested negative [5]. However, this study was limited by the inability to differentiate between asymptomatic and symptomatic infection nor the severity of disease [5]. Alternatively, other researchers have identified severe sequelae of COVID-19 infection in gravid and postpartum patients, such as superimposed bacterial infections due to direct mucosal injury, dysregulation of immune responses, and alterations to the respiratory microbiome after viral pneumonia [6]. Furthermore, the INTERCOVID Multinational Cohort Study has demonstrated that COVID-19-positive gravid women may have a mortality risk that is 22 times greater than uninfected gravid women [7]. Severe pregnancy complications included preeclampsia; eclampsia; hemolysis, elevated liver enzyme, and low platelet (HELLP) syndrome; intensive care unit (ICU) admission or referral to a higher level of care; infections requiring antibiotics; preterm birth; and low birth weight [7]. However, one weakness of this study was that the participants in this study were not risk-stratified to see if they were at an elevated risk of obstetric complications due to their past medical history.

Unfortunately, there are few studies available that analyzed the effect of SARS-CoV-2 on mothers in the postpartum period. One study from Brazil demonstrated a 33.33% rate of ICU admission and a 15.5% mortality rate in postpartum women from complications of COVID-19 infection. This same study showed an ICU admission rate of 19.4% and a mortality rate of 6.3% in pregnant women [8]. While this study demonstrates increased rates of ICU admissions and mortality for COVID-19-infected pregnant women, multiple confounding variables hinder the objective interpretation of this data.

This case report was previously presented as a poster at the Fall 2021 Florida Chapter of the American College of Physicians Hybrid Poster Competition from September 20, 2021, to October 1, 2021.

## Case presentation

A 29-year-old G3P2 female at 30 weeks of gestation presented to the emergency department with a chief complaint of dyspnea and chest pain for two days. This patient had a body mass index of 36.69, and other than two previous caesarean sections, she had no pertinent past medical history or past surgical history. She stated that the dyspnea had gradually worsened since its onset. She reported cough, fever, and myalgias. She endorsed feeling ill for the past week and tested positive for COVID-19 two days prior. The patient denied receiving COVID-19 vaccination. Upon admission, the patient was evaluated by the obstetrics team and was found to have non-reassuring fetal tracings and fetal bradycardia. An urgent caesarean section was performed, and a male neonate was delivered. The appearance, pulse, grimace, activity, and respiration (APGAR) scores were six and seven at one and five minutes, respectively. The baby was transferred to the neonatal intensive care unit (NICU). During the caesarean section, the patient was put under general anesthesia and intubated. However, she was subsequently unable to be extubated. She was admitted to the intensive care unit (ICU) where she was extubated within 24 hours post caesarean section. Infectious disease and critical care were consulted at this time.

**Figure 1:**
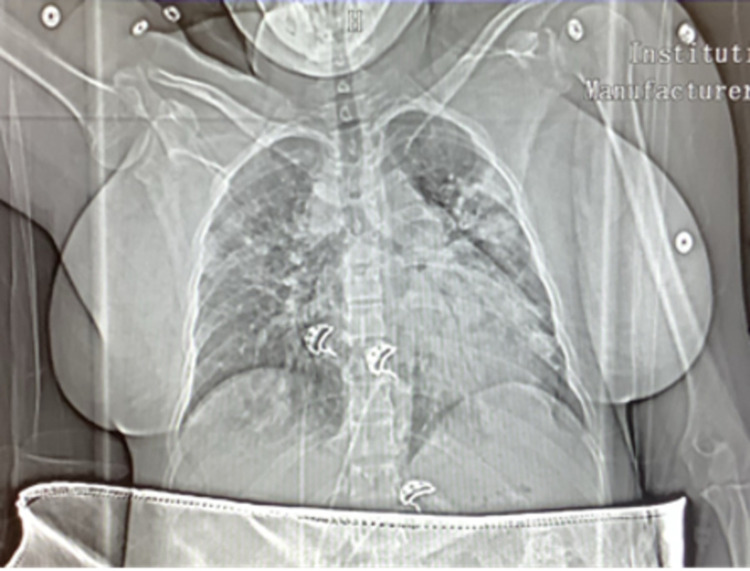
CT angiogram chest showing no evidence of pulmonary emboli and extensive patchy multifocal pneumonia consistent with COVID-19 pneumonia.

**Figure 2:**
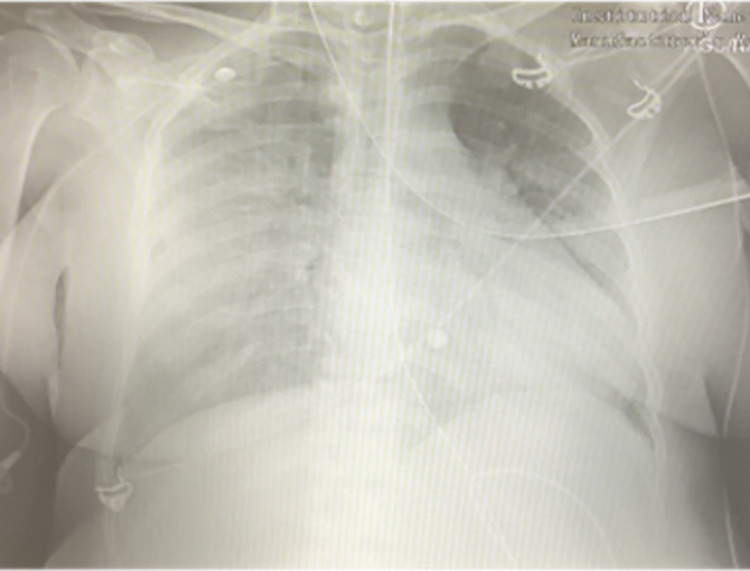
Chest X-ray showing hazy infiltrates with airspace disease throughout the right lung as well as in the left middle and lower lung consistent with viral pneumonia.

After extubation, the patient was placed on a 4 L/minute O_2_ nasal cannula (NC). She reported symptomatic improvement but experienced cough and dyspnea with minimal exertion. Chest computed tomography (CT) showed extensive bilateral pulmonary infiltrates consistent with COVID-19 pneumonia but did not show evidence of pulmonary embolism (Figure 1). On postoperative day one, the patient was transferred to the medical floor due to improved respiratory status. On postoperative day two, she was desaturated to 75% oxygenation, and she was placed on a non-rebreather (NRB) mask with 15 L/minute O_2_ and transferred back to the ICU. Chest X-ray (CXR) revealed severe bilateral airspace disease that had worsened from prior imaging (Figure 2). Dexamethasone and remdesivir treatment were initiated. She was started on a high-flow nasal cannula (HFNC) 50 L/minute at 50% O_2_, and oxygen saturation improved to 93%-96%. She remained tachypneic with a respiratory rate of 36-51 breaths/minute. She was afebrile and normotensive. On postoperative day four, weaning off HFNC was attempted, but she desaturated to 77%, and oxygen support continued. She continued to ambulate minimally due to exertional dyspnea. On postoperative day five, she became tachypneic and anxious. She felt uncomfortable with HFNC and was switched to bilevel positive airway pressure (BiPAP). Oxygen saturation was maintained at around 90%. By postoperative day seven, the patient had a slight improvement in her clinical condition, as she was able to be weaned off BiPAP onto an NRB mask. Additionally, she reported an improvement in her dyspnea. She was also diagnosed with a urinary tract infection (UTI) at this time as her urine culture showed the presence of gram-negative lactose-fermenting bacilli; she was started on ceftriaxone.

On postoperative day nine, the patient’s condition worsened overnight, with oxygen desaturation of about 80% and temperature of 100.1°F. She was placed on BiPAP again. She also reported having anxiety from mask placement. On postoperative day 10, she was intubated with sedation due to worsening respiratory status. She became hypotensive and developed a third-degree heart block. Subsequently, she experienced cardiac arrest with a return of spontaneous circulation (ROSC) in nine minutes. External cardiac pacing and pressors were started. Subsequently, she had seizure activity and was given intravenous lorazepam. On postoperative day 12, she did not have a cough or gag reflex to suction, and her pupils were nonreactive to light. Electroencephalography (EEG) performed shortly after decreasing sedation revealed burst suppression activity. We suspected that her EEG findings were due to hypoxic encephalopathy secondary to COVID-19 pneumonia and cardiorespiratory arrest. Repeat EEG two days later showed marked diffuse symmetrical slowing that confirmed hypoxic-ischemic encephalopathy. After several days of discussion with family members, it was determined that the patient would require long-term cardiorespiratory and nutritional support. Due to this, a percutaneous endoscopic gastrostomy (PEG) tube was placed along with a tracheostomy tube because she lacked the ability to breathe spontaneously without a ventilator. Unfortunately, after approximately nine weeks of life support, the patient was transferred to hospice care due to complications secondary to hypoxic encephalopathy and cardiorespiratory arrest. The baby was discharged home after observation in the NICU and is currently doing well.

## Discussion

This case represents an important example of how COVID-19 infection affects the respiratory health of young, unvaccinated, gravid, and postpartum patients. This patient initially presented in a stable condition to the emergency department and subsequently experienced a decline in her respiratory status after caesarean section. What makes this patient’s case interesting is that she had no known risk factors for severe COVID-19 infection. While the patient was displaying early symptoms of COVID-19 infection upon admission, any link between maternal COVID-19 infection and fetal bradycardia is not currently well described in the scientific literature. Limited studies have found an association between maternal COVID-19 infection and other changes in fetal heart tracing (FHT). One study from Spain showed that symptomatic COVID-19 infection can cause late or prolonged decelerations, loss of accelerations, and increases in baseline fetal heart rate [9]. Despite the development of a category III FHT in this patient, the caesarean delivery was successful without fetal demise. Also, studies have shown that while pregnant patients are less likely to experience expected symptoms such as muscle aches, fever, and chills from COVID-19, they are more likely to suffer respiratory decline [10].

While our patient was experiencing symptoms of COVID-19 infection before delivery, her respiratory status began to deteriorate in the postoperative setting. An important question to consider is whether the physiological changes in maternal immunity as a result of pregnancy had an impact on the progression of the infection. Maternal immunological changes of pregnancy include attenuation in adaptive immune function efficiency, including less efficacious infection-fighting, which are most pronounced during the third trimester [11]. Furthermore, functional residual capacity (FRC) and total respiratory compliance are decreased, most notably during the third trimester [11]. In addition, it is likely that the caesarean section had an appreciable effect on disease progression, as major abdominal surgeries are associated with significant systemic physiological stress. The typical response to surgery includes increased levels of cortisol and growth hormone synthesis that can induce a hypermetabolic and hypercatabolic state, leading to muscle wasting, impaired immune function, and organ failure [12]. These immune system adaptations during pregnancy and the postpartum period may have contributed to the clinical progression of the patient’s COVID-19 infection. While it is impossible to comment on whether she would have survived the complications of COVID-19 infection if she had not been infected while pregnant, the combination of immunocompromised state of pregnancy and major abdominal surgery likely contributed to her demise. This underscores how vital it is for pregnant patients to mitigate exposure to COVID-19 and be vaccinated. COVID-19 vaccines are safe in pregnancy [13].

## Conclusions

COVID-19 infection poses significant life-threatening risks for gravid patients including, but not limited to, acute respiratory distress syndrome, pneumonia, respiratory failure, hypoxic encephalopathy, coma, and sudden cardiac death. Current research is primarily focused on the effect of COVID-19 infection on gravid patients during the prenatal period, with limited data during the peripartum and postpartum period. This case highlights the need for additional research into the short- and long-term effects of SARS-CoV-2 infection during the peripartum and postpartum periods to develop guideline-directed medical therapy for the management of these patients. Furthermore, it shows the importance of COVID-19 vaccination for women who are pregnant or plan to become pregnant to reduce the risk of contracting the virus and experiencing its life-threatening sequelae.
